# Early prediction of late-pregnancy hypertriglyceridemia in women with gestational diabetes: development and internal validation of a clinical risk model

**DOI:** 10.3389/fendo.2026.1845573

**Published:** 2026-06-26

**Authors:** Yaping Zhao, Juanjuan Zhang, Xiaoyan Jin

**Affiliations:** Department of Obstetrics and Gynecology, Affiliated Kunshan Hospital of Jiangsu University, Kunshan, Jiangsu, China

**Keywords:** gestational diabetes mellitus, hypertriglyceridemia, prediction model, pregnancy, risk stratification

## Abstract

**Background:**

Women with gestational diabetes mellitus (GDM) are at high risk of developing hypertriglyceridemia (HTG) in late pregnancy, a condition associated with adverse maternal and neonatal outcomes. Early identification of high-risk individuals at GDM diagnosis is crucial for timely intervention.

**Objective:**

To develop and internally validate a clinical risk prediction model for estimating the individualized risk of late-pregnancy HTG in women at the time of GDM diagnosis.

**Methods:**

This single-center retrospective cohort study included 587 women with GDM. Predictor variables, available at or before GDM diagnosis (24–28 weeks), included demographic characteristics, glycemic markers from the 75g oral glucose tolerance test (OGTT), and first-trimester lipid profiles. The outcome was late-pregnancy HTG, defined as a triglyceride level ≥ 2.3 mmol/L. The dataset was randomly split into a training set (70%) and a held-out internal test set (30%) for secondary performance assessment, whereas bootstrap resampling of the full analytic cohort was used as the primary internal validation strategy to quantify optimism. Variable selection was performed using LASSO logistic regression with 10-fold cross-validation in the training set, followed by standard multivariable logistic regression. Model performance was assessed using discrimination, calibration-in-the-large, calibration slope, Brier score, scaled Brier score, and decision curve analysis.

**Results:**

The incidence of late-pregnancy HTG was 32.7% (192/587). The final model incorporated five predictors: pre-gravid BMI, fasting plasma glucose (FPG) at diagnosis, 1-hour post-load glucose (1hPG) at diagnosis, first-trimester triglycerides (TG), and first-trimester high-density lipoprotein cholesterol (HDL-C). The model demonstrated good discrimination in the held-out internal test set, with an AUC of 0.816 (95% CI: 0.754–0.878), calibration-in-the-large of −0.03, calibration slope of 0.94, Brier score of 0.151, and scaled Brier score of 0.301. Bootstrap internal validation of the full cohort showed limited optimism, with an optimism-corrected AUC of 0.805, calibration-in-the-large of −0.02, calibration slope of 0.91, Brier score of 0.158, and scaled Brier score of 0.269. Decision curve analysis supported clinical utility across clinically relevant thresholds, and the full five-variable model provided modestly higher net benefit than the four-variable model excluding first-trimester TG.

**Conclusion:**

This study developed and internally validated a prediction model for late-pregnancy HTG in women with GDM using five routinely available clinical parameters. The model shows promising performance for individualized risk stratification at GDM diagnosis, potentially facilitating targeted monitoring and management. External validation in independent multicenter cohorts is required before clinical implementation.

## 1 Introduction

Gestational Diabetes Mellitus (GDM) is one of the most common metabolic complications in pregnancy, and its global prevalence is continuously rising, posing an increasingly severe challenge to maternal and infant health (1). The core pathophysiological mechanism of GDM lies in the progressive development of insulin resistance, a state that not only disrupts the mother’s glucose metabolism but also profoundly affects lipid metabolism (2). In the GDM environment, decreased insulin sensitivity leads to accelerated lipolysis in adipose tissue, releasing excessive free fatty acids into circulation. The liver utilizes this surplus of substrates, exacerbating the synthesis and secretion of very-low-density lipoprotein (VLDL), ultimately manifesting as a significant elevation in serum triglyceride (TG) levels, known as hypertriglyceridemia (HTG) (3). Therefore, HTG is not merely a concomitant phenomenon of GDM but a core manifestation of its pathophysiological changes within the realm of lipid metabolism.

The emergence of HTG in mid-to-late pregnancy is not a harmless biochemical fluctuation but is closely associated with a series of adverse maternal and neonatal outcomes. A substantial body of research has confirmed that elevated TG levels in the third trimester of GDM patients are an independent risk factor for macrosomia, large for gestational age (LGA) infants, and preterm birth (4). More alarmingly, when TG levels rise to extremely high concentrations (e.g., >11.3 mmol/L), it can trigger hypertriglyceridemia-induced acute pancreatitis (HTG-AP), a highly dangerous obstetric emergency that poses a direct and severe threat to the lives of both mother and baby (5). Although the potential dangers of HTG are clinically recognized, the current practical dilemma is that lipid management for GDM patients is often reactive, with interventions initiated only after significant lipid abnormalities are detected late in pregnancy. There is a general lack of a prospective, early risk stratification tool that can be implemented at the time of GDM diagnosis in mid-pregnancy. This situation highlights a critical unmet need in the current clinical management paradigm.

Currently, significant progress has been made in the research of clinical prediction models for GDM, but the vast majority of these models focus on predicting the risk of developing GDM itself (6). Although some studies have explored the cross-sectional relationship between GDM and the lipid profile, to our knowledge, there is currently no clinical prediction model specifically designed for the cohort of patients already diagnosed with GDM, that can prospectively and quantitatively predict their risk of developing HTG in late pregnancy at the time of diagnosis (24–28 weeks of gestation). This gap in the existing literature means that clinicians managing GDM patients lack an effective tool to identify high-risk individuals who may develop severe lipid metabolism disorders in the future, thus precluding the implementation of precise, individualized monitoring and preventive strategies. Therefore, risk stratification for late-gestational HTG at the critical clinical window of GDM diagnosis is an important and urgent knowledge gap to be filled.

To address the aforementioned challenges and fill this knowledge gap, this study aims to develop and internally validate a multivariable clinical prediction model. The model is designed to predict the individualized risk of late-gestational HTG in GDM patients at the time of diagnosis, using routinely collected data such as demographic characteristics, obstetric history, oral glucose tolerance test (OGTT) results, and early-pregnancy lipid levels. The ultimate goal of this research is to provide a practical and convenient quantitative tool, with the aim of furnishing an evidence-based foundation for individualized, stratified lipid management and early intervention for GDM patients, thereby improving maternal and infant outcomes.

## 2 Methods

### 2.1 Study design

This single-center retrospective cohort study was conducted using electronic medical records (EMR) to develop and internally validate a clinical risk model for predicting the development of late-pregnancy HTG in women diagnosed with GDM. Data were collected from Affiliated Kunshan Hospital of Jiangsu University for the period from January 1, 2022, to June 30, 2025. The study adhered to the TRIPOD (Transparent Reporting of a multivariable prediction model for Individual Prognosis Or Diagnosis) guidelines. Time zero was explicitly defined as the date of GDM diagnosis, typically occurring between 24 and 28 weeks of gestation, ensuring that all predictor variables were obtained at or prior to this time point. The study protocol was reviewed and approved by the Ethics Committee of Affiliated Kunshan Hospital of Jiangsu University. A waiver of informed consent was granted due to the retrospective nature of the study and the use of fully anonymized data, in accordance with local regulatory standards and the principles of the Declaration of Helsinki. All data management and analysis procedures were performed with strict confidentiality and privacy protection measures.

### 2.2 Study population

The study cohort was identified through a systematic query of the institutional EMR system. All pregnant women with a recorded diagnosis of GDM during the study period (January 1, 2022, to June 30, 2025) were initially screened. Patient identification and eligibility confirmation were performed through a two-stage process: (1) automated extraction using diagnostic codes and laboratory data filters, followed by (2) a detailed, manual review of individual medical records by two independent clinical researchers to verify all inclusion and exclusion criteria based on structured data entry.

Inclusion criteria comprised all of the following: (1) Singleton pregnancy; (2) Diagnosis of GDM confirmed between 24 and 28 weeks of gestation via a 75g oral glucose tolerance test (OGTT) according to the International Association of the Diabetes and Pregnancy Study Groups (IADPSG) criteria (7), defined as meeting or exceeding at least one of the following plasma glucose thresholds: fasting ≥ 5.1 mmol/L, 1-hour ≥ 10.0 mmol/L, or 2-hour ≥ 8.5 mmol/L; (3) Availability of at least one valid fasting lipid measurement, including triglycerides, performed within the predefined late-pregnancy outcome-assessment window of 32 + 0 to 36 + 6 gestational weeks.

Exclusion criteria encompassed any of the following conditions, ascertained from medical records prior to or at the time of GDM diagnosis unless otherwise specified: (1) Pre-existing diabetes mellitus (Type 1 or Type 2) (1, 8); (2) Known history of pre-gestational dyslipidemia, chronic hypertension, or cardiovascular disease (1, 8); (3) Multiple gestation (e.g., twins or higher-order pregnancies) (1, 8); (4) Comorbid endocrine disorders (e.g., thyroid dysfunction) or severe hepatic/renal insufficiency that could secondarily affect lipid metabolism (8, 9); (5) Use of medications known to significantly influence glucose or lipid metabolism during pregnancy (e.g., statins, systemic corticosteroids) (1); (6) Critical missing data for key predictor variables essential for model development, such as pre-pregnancy weight or glycemic values at GDM diagnosis (8); and (7) Failure to establish late-pregnancy HTG status within the predefined outcome-assessment window was also an exclusion criterion. This included two non-overlapping categories: women who otherwise remained under care but had no qualifying fasting lipid measurement between 32 + 0 and 36 + 6 gestational weeks, and women whose outcome assessment could not be completed because they were transferred to another institution, were lost before assessment, or had not reached the outcome window before the data collection cut-off date of June 30, 2025.

### 2.3 Variable definitions and data collection

The primary outcome of this study was late-pregnancy HTG, defined as a binary variable based on fasting serum TG measured within a predefined gestational age window of 32 + 0 to 36 + 6 weeks. There is no universally accepted pregnancy-specific TG threshold for defining clinically relevant HTG in late pregnancy. Therefore, the primary threshold of TG ≥ 2.3 mmol/L (203.5 mg/dL) was selected as a pragmatic definition of moderate late-pregnancy TG elevation, rather than as a threshold for severe HTG or HTG-induced acute pancreatitis. This threshold is above the conventional non-pregnant adult threshold of 1.7 mmol/L and is close to pregnancy-related TG cut-offs reported in studies examining adverse pregnancy outcomes (9, 10). To examine whether model performance was sensitive to the selected outcome threshold, additional sensitivity analyses were performed using TG cut-offs of ≥1.7, ≥2.5, and ≥2.83 mmol/L. For women with more than one fasting lipid panel within this window, the measurement closest to 34 + 0 gestational weeks was selected as the outcome-defining value. Women who did not have an outcome-defining lipid measurement within this window before the data collection cut-off were not included in the analytic cohort, because their late-pregnancy HTG status could not be reliably classified. All predictor variables were collected at or before GDM diagnosis and therefore preceded the outcome lipid measurement.

A comprehensive set of predictor variables was selected based on established pathophysiological mechanisms of dyslipidemia in GDM and a thorough literature review. All data were extracted retrospectively from the institutional EMR system. Baseline demographic and clinical characteristics included maternal age at delivery, pre-gravid weight and height, parity (number of previous live births), and gravidity (total number of pregnancies). Pre-gravid Body Mass Index (BMI) was calculated as weight (kg)/height (m)² and categorized according to World Health Organization criteria (11). Pre-pregnancy weight was primarily based on self-report recorded at the first prenatal visit. Because self-reported pre-pregnancy weight may be systematically underestimated, the first measured antenatal weight was also extracted and used for agreement assessment and sensitivity analysis. Obstetric history, including a history of prior GDM or delivery of a macrosomic infant (>4000g), along with a documented family history of diabetes in a first-degree relative, was also recorded.

Key clinical data at the time of GDM diagnosis were collected. These included the results of the 75g OGTT: fasting plasma glucose (FPG), 1-hour post-load glucose (1hPG), and 2-hour post-load glucose (2hPG). Glycated hemoglobin (HbA1c) levels recorded at the time of GDM diagnosis were also extracted. Gestational weight gain (GWG) up to GDM diagnosis was calculated as the difference between the weight recorded at the time of the OGTT and the pre-gravid weight. To account for baseline metabolic status, the earliest first-trimester (≤13 weeks gestation) fasting lipid profile was used, including TG, total cholesterol (TC), high-density lipoprotein cholesterol (HDL-C), and low-density lipoprotein cholesterol (LDL-C). Because first-trimester lipid indices were prespecified candidate predictors for model development, patients without any available first-trimester fasting lipid profile were excluded before construction of the analytic cohort. Management strategy for GDM following diagnosis was categorized as diet and lifestyle modification only, metformin, or insulin therapy.

### 2.4 Sample size and data processing

As this was a retrospective cohort study, the sample size was determined by the number of eligible women with GDM during the predefined study period. We therefore assessed the adequacy of the available sample for prediction-model development using the events-per-variable (EPV) principle, with an EPV ≥ 10 considered the minimum criterion to reduce the risk of model overfitting (12). In this study, an event was defined as late-pregnancy hypertriglyceridemia (HTG). The anticipated event frequency was considered in relation to published data from comparable GDM cohorts using triglyceride thresholds close to the present study definition.

A key study by Lai et al. (2020) reported that among 513 GDM patients, 33.3% had TG levels within the range of 2.14–2.89 mmol/L during their 24th-28th gestational weeks, a timeframe and value range that closely aligns with our diagnostic criteria (13). Furthermore, research from Puga et al. (2024) observed that with a slightly lower cutoff of ≥1.7 mmol/L, the incidence of HTG in late pregnancy (32–36 weeks) was as high as 50.8% (14). This is strongly supported by physiological data from multiple meta-analyses showing that the average third-trimester TG level in GDM patients is approximately 2.36-2.55 mmol/L. On this basis, an event rate of approximately one-third was considered clinically plausible for evaluating model-development feasibility. The final cohort included 587 women, among whom 192 developed late-pregnancy HTG, yielding approximately 12.8 to 19.2 events per candidate predictor for an initial set of 10 to 15 candidate variables. Thus, the available sample satisfied the commonly used EPV ≥ 10 criterion and was considered adequate for developing a parsimonious prediction model with overfitting control through LASSO selection and bootstrap internal validation.

Data were extracted from the institutional EMR system using structured queries with predefined logic checks for consistency and range validation. A rigorous two-phase data validation process was implemented: (1) automated extraction with built-in validation rules to identify outliers and inconsistencies; and (2) manual chart review of a randomly selected 15% audit sample by two trained clinical researchers to verify data accuracy. Specifically, 88 of the 587 eligible records were independently re-reviewed. Inter-rater agreement was assessed for key categorical or abstraction-dependent variables, including GDM diagnosis based on 75-g OGTT criteria, history of prior GDM, family history of diabetes, GDM management strategy, and late-pregnancy HTG status. The corresponding Cohen’s κ values were 0.96, 0.92, 0.91, 0.94, and 0.98, respectively. Overall, 9 records had at least one discrepant item between the two reviewers and were adjudicated through joint case review with a senior obstetric clinician. Continuous laboratory variables were extracted directly from the central laboratory database and were verified using range and temporal consistency checks rather than κ statistics. Baseline demographic information, obstetric history, and family history were ascertained from standardized prenatal intake forms. Laboratory values, including results from the 75-g OGTT, HbA1c, and all lipid profiles, were obtained from the hospital’s central laboratory database. Pre-pregnancy weight was primarily sourced from self-report documented at the first prenatal visit. The first measured antenatal weight was available for 552 of 587 women (94.0%), at a median gestational age of 9.6 weeks (interquartile range, 8.4–11.2 weeks). Agreement between self-reported pre-pregnancy weight and first measured antenatal weight was evaluated using mean difference, Pearson correlation, intraclass correlation coefficient (ICC), and Bland–Altman limits of agreement. All data points were required to be temporally aligned with the study definition, ensuring predictors were recorded at or before the GDM diagnosis timepoint. Missingness was first summarized for each prespecified candidate predictor before imputation. Among the 952 potential participants assessed after initial eligibility screening, 365 were excluded before construction of the final analytic cohort. These included 100 women with missing essential baseline data required for model development, comprising absence of any first-trimester fasting lipid profile (n = 72, 7.6%) or unavailable pre-gravid BMI information (n = 28, 2.9%); 201 women without a qualifying fasting lipid measurement within the predefined 32 + 0 to 36 + 6-week outcome-assessment window; and 64 women whose outcome ascertainment could not be completed because of transfer or loss before assessment or because they had not reached the outcome window before the data collection cut-off date. Therefore, 587 women were included in the final analytic cohort. In the final analytic cohort of 587 women, the outcome variable, OGTT-derived glucose values, pre-gravid BMI, and first-trimester lipid components were complete after eligibility screening. Missingness remained low for several non-essential candidate predictors: HbA1c at GDM diagnosis, 46/587 (7.8%); gestational weight gain up to GDM diagnosis, 21/587 (3.6%); family history of diabetes, 18/587 (3.1%); and history of prior GDM, 7/587 (1.2%). No other candidate predictor had missingness exceeding 1%. Because missingness was mainly related to the timing and completeness of routine prenatal documentation rather than the subsequently measured HTG outcome, the missing-data mechanism was assumed to be missing at random (MAR). Multiple imputation by chained equations was therefore performed to generate 10 imputed datasets, including all candidate predictors and the outcome variable in the imputation model (15).

### 2.5 Statistical analysis

All statistical analyses were performed using R software (version 4.3.0). Descriptive statistics were used to summarize the baseline characteristics of the overall cohort and to compare these characteristics between women who developed late-pregnancy HTG and those who did not. Continuous variables were presented as mean ± standard deviation or median (interquartile range) based on their distribution, assessed using the Shapiro-Wilk test, and compared using the Student’s t-test or Mann-Whitney U test, as appropriate. Categorical variables were expressed as numbers (percentages) and compared using the Chi-square test or Fisher’s exact test. The gestational age at outcome lipid assessment was summarized as median and interquartile range and compared between women with and without late-pregnancy HTG to evaluate temporal comparability of outcome measurement. For prediction model development, all continuous predictor variables were standardized using Z-score normalization prior to analysis. The complete dataset was randomly split into a training set (70%) for model development and a held-out internal test set (30%) for secondary performance assessment, ensuring that the proportion of HTG outcomes was similar in both sets via stratified sampling. Because random split-sample validation can reduce the effective sample size for model development and may yield unstable performance estimates in moderate-sized datasets, the 7:3 split was retained only as a secondary, transparent assessment of model performance within the same source population. The primary internal validation procedure was bootstrap optimism correction using the full analytic cohort, which is generally recommended for prediction-model development. Variable selection and model construction were conducted exclusively within the training set. To integrate multiple imputation with penalized variable selection, LASSO logistic regression with 10-fold cross-validation was performed independently in each of the 10 imputed training datasets. In each imputed dataset, the optimal penalty parameter (λ) was selected using lambda.min, and variables with non-zero coefficients at this λ were recorded. Because variable selection after multiple imputation may vary across imputed datasets, a prespecified selection-frequency rule was used: predictors selected in at least 6 of the 10 imputed datasets were retained for the final model. The complete list of candidate predictors, their missingness rates, and variance inflation factors (VIFs) were summarized to improve transparency of model specification and to evaluate multicollinearity among candidate variables. VIFs were calculated using the candidate predictor set before penalized regression; a VIF < 5 was considered to indicate no severe multicollinearity. To further assess variable-selection stability, bootstrap inclusion frequency was calculated using 1000 patient-level bootstrap replicates. In each bootstrap replicate, the complete modeling workflow was repeated, including multiple imputation, standardization, LASSO selection, and application of the same selection-frequency rule across imputed datasets. The proportion of bootstrap replicates in which each candidate predictor was retained was reported as the bootstrap inclusion frequency. For enhanced clinical interpretability, the selected predictors were then entered into standard unpenalized multivariable logistic regression models fitted separately in the 10 imputed training datasets, and regression coefficients and standard errors were pooled using Rubin’s rules. This final model was used to report the regression coefficients (β), adjusted odds ratios (ORs), and their corresponding 95% confidence intervals (CIs) for each predictor. Model performance was rigorously evaluated. Discrimination, the model’s ability to distinguish between women with and without HTG, was assessed in the held-out internal test set by calculating the C-statistic (Area Under the Receiver Operating Characteristic Curve, AUC) along with its 95% CI, and visualized using an ROC curve. Calibration, the agreement between predicted probabilities and observed outcomes, was evaluated in the held-out internal test set using a calibration plot, calibration-in-the-large, calibration slope, and the Hosmer-Lemeshow goodness-of-fit test. Calibration-in-the-large was estimated as the intercept from a logistic recalibration model with the linear predictor offset, and the calibration slope was estimated by regressing the observed outcome on the model linear predictor. Decile-level calibration was further assessed by grouping patients in the held-out internal test set into 10 groups according to predicted risk and comparing the mean predicted probability with the observed event proportion in each group.

**Figure 1:**
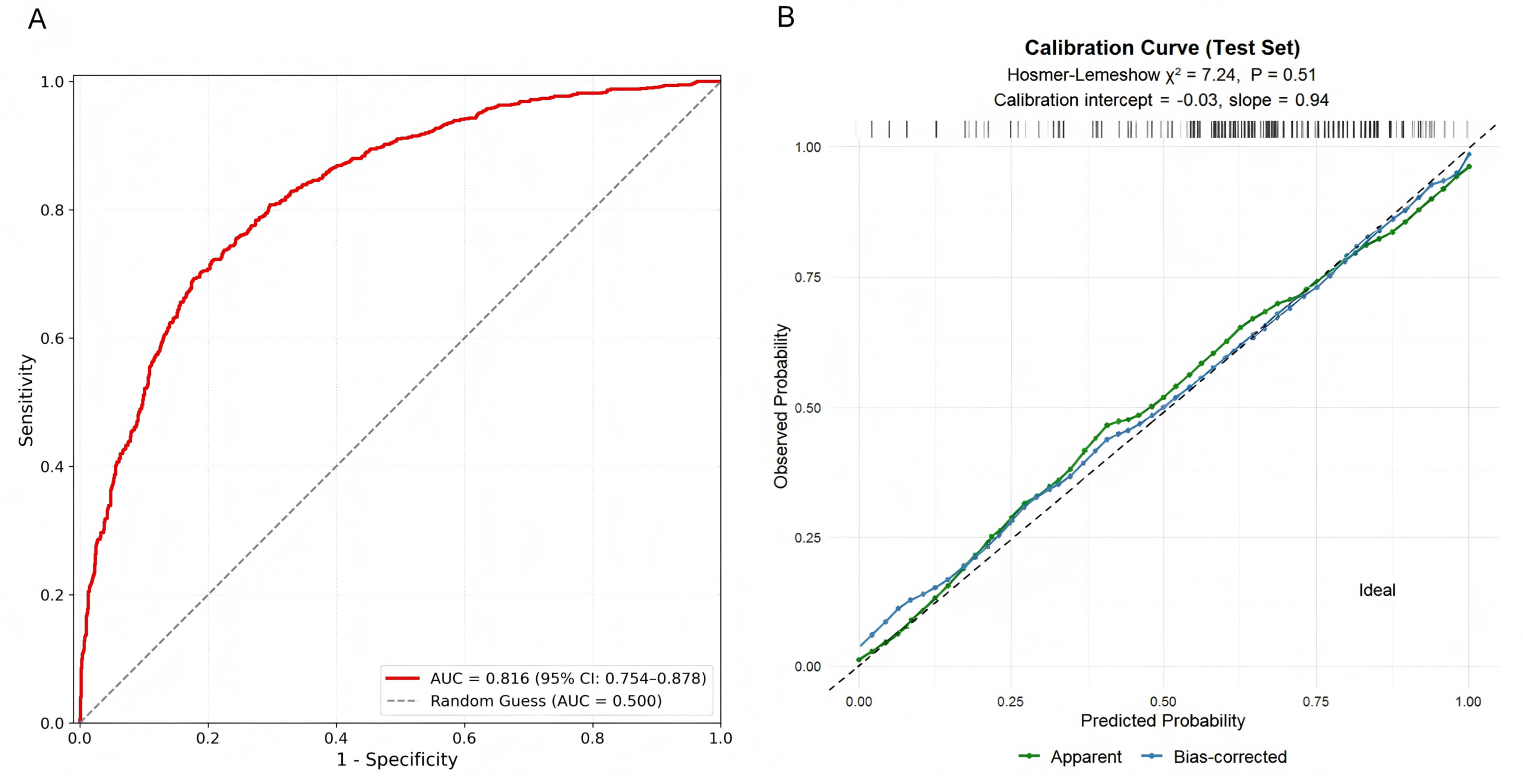
Discrimination and calibration performance of the prediction model. **(A)** Receiver operating characteristic (ROC) curve of the prediction model in the held-out internal test set, showing an area under the curve (AUC) of 0.816 (95% CI: 0.754–0.878). The dashed diagonal line represents random classification performance. **(B)** Calibration curve assessing agreement between predicted probabilities and observed outcomes. The green line represents the apparent calibration curve in the held-out internal test set, and the blue line represents the bootstrap optimism-corrected calibration curve generated from 1000 patient-level resamples of the full analytic cohort. The dashed diagonal line indicates ideal calibration, and the rug plot at the top shows the distribution of predicted probabilities. In the held-out internal test set, the calibration-in-the-large was −0.03, the calibration slope was 0.94, and the Hosmer-Lemeshow goodness-of-fit test showed no significant lack of fit (χ² = 7.24, P = 0.510). The corresponding bootstrap optimism-corrected calibration-in-the-large and calibration slope were −0.02 and 0.91, respectively.

To assess optimism, 1000 patient-level bootstrap resamples were generated from the full analytic cohort. In each resample, the complete modeling process was repeated, including imputation, standardization, LASSO selection, final logistic regression, and performance estimation. Optimism-corrected estimates were obtained for the C-statistic, calibration-in-the-large, calibration slope, Brier score, and scaled Brier score. The bootstrap-corrected calibration curve in **Figure 1B** was generated using the same procedure. For clinical application, a nomogram was constructed based on the coefficients from the final standard logistic regression model to provide a visual tool for calculating individualized HTG risk. To evaluate the incremental value of first-trimester TG and the full five-variable model, three reference models were constructed and evaluated using the same training and held-out internal test sets: first-trimester TG alone, pre-gravid BMI plus first-trimester TG, and a four-variable model excluding first-trimester TG but retaining pre-gravid BMI, FPG at GDM diagnosis, 1hPG at GDM diagnosis, and first-trimester HDL-C. Discrimination, calibration, Brier score, classification performance, and clinical utility were compared across the reference models and the full five-variable model. Pairwise AUC comparisons were performed using DeLong’s test, and clinical utility was compared using decision curve analysis. In addition to the probability threshold selected by Youden’s J statistic, classification performance was evaluated at prespecified clinically meaningful risk thresholds of 0.10, 0.20, 0.30, 0.40, and 0.50, including sensitivity, specificity, PPV, NPV, and accuracy. Finally, Decision Curve Analysis (DCA) was performed in the held-out internal test set to evaluate clinical utility across threshold probabilities from 0.10 to 0.50. Net benefit was compared with the default strategies of intervening for all patients and intervening for no patients. Three sensitivity analyses were performed. First, gestational age at outcome lipid measurement was additionally included in the final model to evaluate whether residual variation in measurement timing within the predefined 32 + 0 to 36 + 6-week outcome window influenced model performance. The five predictors selected in the primary model were retained, and gestational age at outcome lipid measurement was added as an additional covariate. The sensitivity model was fitted using the same training-set procedure as the primary model and evaluated in the held-out internal test set. Discrimination was assessed using the AUC, calibration was assessed using the calibration curve and Hosmer-Lemeshow goodness-of-fit test, and classification performance was evaluated using sensitivity, specificity, PPV, and NPV at the same probability threshold used for the primary model. Second, additional sensitivity analyses were conducted to assess whether model performance was robust to the TG threshold used to define late-pregnancy HTG. Within the same predefined 32 + 0 to 36 + 6-week outcome window, alternative TG cut-offs of ≥1.7, ≥2.5, and ≥2.83 mmol/L were applied. For each alternative outcome definition, the same five-predictor model structure was refitted in the training set and evaluated in the held-out internal test set using AUC, calibration, sensitivity, specificity, PPV, and NPV. Third, to examine whether reliance on self-reported pre-pregnancy weight influenced model performance, a sensitivity analysis was performed among women with available first measured antenatal weight. Pre-gravid BMI in the final five-predictor model was replaced by BMI calculated from first measured antenatal weight, while the other four predictors were unchanged. The model was refitted in the training set and evaluated in the held-out internal test set using AUC, calibration slope, calibration-in-the-large, Brier score, sensitivity, specificity, PPV, and NPV.

## 3 Results

### 3.1 Study cohort characteristics

**Figure 2:**
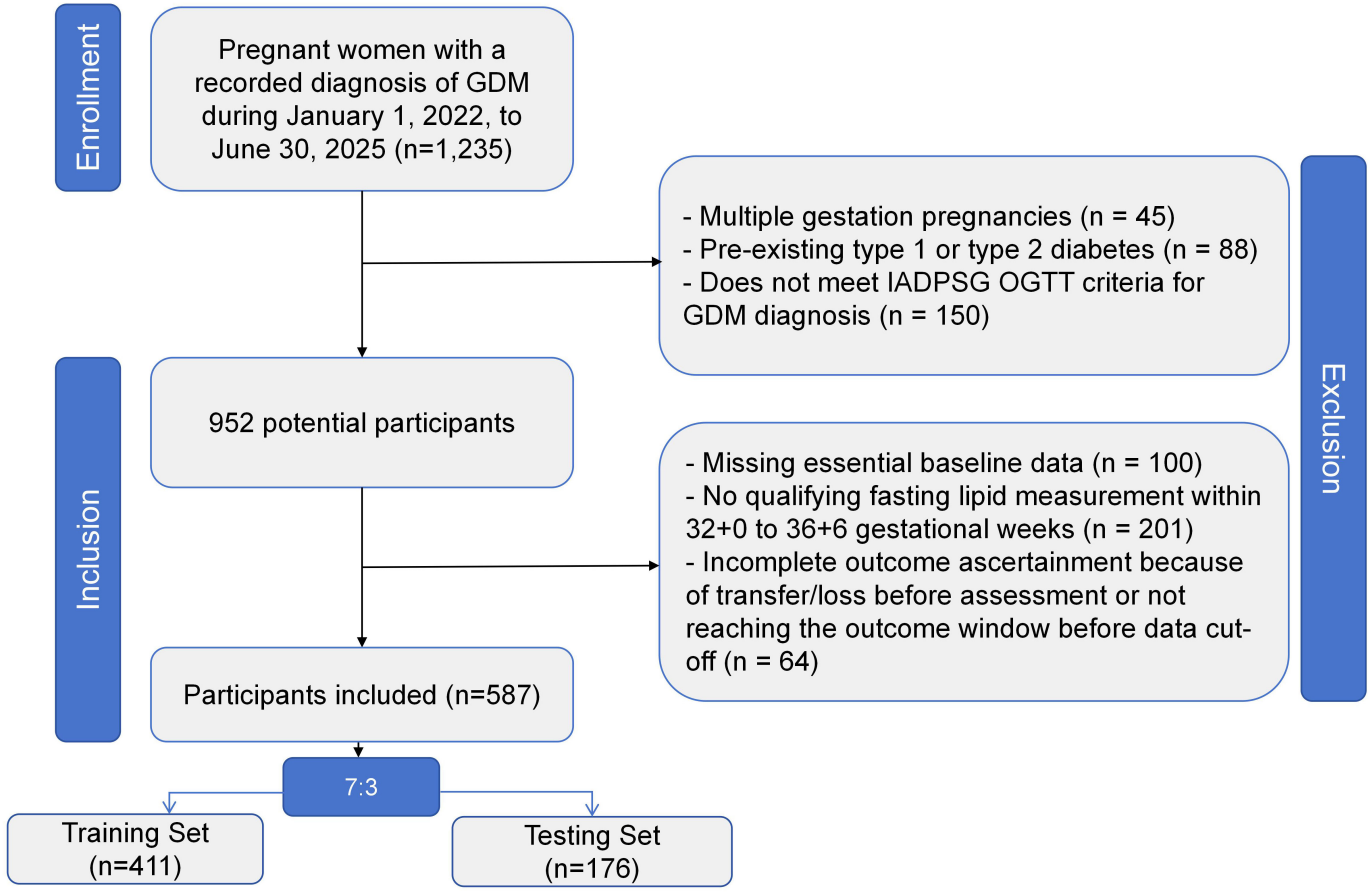
Inclusion and exclusion flowchart.

The patient selection process is detailed in **Figure 2**. Initially, 1,235 pregnant women with a recorded diagnosis of GDM during the study period were identified. After applying the exclusion criteria, 587 women constituted the final study cohort. After initial screening, 1,235 pregnant women with a recorded diagnosis of GDM were identified. A total of 283 women were excluded at the eligibility-confirmation stage, including 45 with multiple gestations, 88 with pre-existing type 1 or type 2 diabetes, and 150 who did not meet the IADPSG OGTT criteria for GDM. Among the remaining 952 potential participants, 365 were further excluded: 100 had missing essential baseline data required for model development, 201 had no qualifying fasting lipid measurement within the predefined 32 + 0 to 36 + 6-week outcome-assessment window, and 64 could not complete outcome ascertainment because of transfer or loss before assessment or because they had not reached the outcome window before the data collection cut-off date. Finally, 587 women constituted the analytic cohort. These patients were not treated as outcome-negative cases because their late-pregnancy HTG status could not be reliably determined. Among the remaining exclusions, 100 women were excluded because of missing essential baseline data required for model development, including absence of any first-trimester fasting lipid profile (n = 72) or unavailable pre-gravid BMI information (n = 28). In the final analytic cohort, the outcome variable, OGTT-derived glucose values, pre-gravid BMI, and first-trimester lipid components were complete. The first measured antenatal weight was available for 552 women (94.0%). Compared with first measured antenatal weight, self-reported pre-pregnancy weight was slightly lower, with a mean difference of −0.8 ± 2.4 kg. The Pearson correlation coefficient was 0.97, and the ICC was 0.96 (95% CI: 0.95–0.97). Bland–Altman analysis showed no major systematic disagreement, with 95% limits of agreement from −5.5 to 3.9 kg. BMI category agreement between self-reported pre-pregnancy BMI and first-visit measured BMI was 91.8%, with a weighted κ of 0.87 (**Supplementary Table 1**). Missingness was limited to HbA1c at GDM diagnosis (46/587, 7.8%), gestational weight gain up to GDM diagnosis (21/587, 3.6%), family history of diabetes (18/587, 3.1%), and history of prior GDM (7/587, 1.2%), as summarized in **Supplementary Table 2**. As shown in **Table 1**, the baseline characteristics of the cohort, stratified by late-pregnancy HTG status, were compared. The overall incidence of late-pregnancy HTG (TG ≥ 2.3 mmol/L) was 32.7% (192/587). The fasting lipid measurement used for outcome assessment was performed at a median gestational age of 34.4 weeks (interquartile range, 33.7–35.2 weeks; range, 32 + 0 to 36 + 6 weeks). The distribution of outcome-assessment timing was 141 women (24.0%) at 32 + 0 to 33 + 6 weeks, 339 women (57.8%) at 34 + 0 to 35 + 6 weeks, and 107 women (18.2%) at 36 + 0 to 36 + 6 weeks. The gestational age at lipid measurement was comparable between the non-HTG and HTG groups [34.4 (33.7–35.2) vs. 34.5 (33.8–35.3) weeks, P = 0.418], supporting temporal comparability of outcome ascertainment between groups. Significant differences were observed between the Non-HTG (n=395) and HTG (n=192) groups. Women who developed HTG were older (33.1 ± 4.3 vs. 30.3 ± 5.0 years, p<0.001), had a higher pre-gravid BMI (27.8 ± 4.9 vs. 24.0 ± 4.2 kg/m², p<0.001), and were more likely to be classified as overweight or obese before pregnancy (65.1% vs. 29.6%, p<0.001). Glycemic control at GDM diagnosis was notably worse in the HTG group, as evidenced by significantly higher FPG, 1hPG, 2hPG, and HbA1c levels (all p<0.001). Furthermore, the HTG group demonstrated a more adverse early-pregnancy lipid profile, with higher first-trimester TG and LDL-C levels, and lower HDL-C levels (all p<0.01). There were no significant differences between the groups in terms of parity, gravidity, history of prior GDM, family history of diabetes, or gestational weight gain up to GDM diagnosis. The management strategy for GDM also differed significantly, with a higher proportion of women in the HTG group requiring pharmacotherapy (metformin or insulin) beyond lifestyle modification (p<0.001).

**Table 1:** Baseline characteristics of the study cohort, stratified by late-pregnancy hypertriglyceridemia status.

Characteristic	Total (N = 587)	Non-HTG group (n=395)	HTG group (n=192)	Statistical value	P-value
Maternal Age (years)	31.2 ± 4.9	30.3 ± 5.0	33.1 ± 4.3	t=6.75	<0.001
Pre-gravid BMI (kg/m²)	25.3 ± 4.8	24.0 ± 4.2	27.8 ± 4.9	t=9.48	<0.001
Pre-gravid BMI Category, n (%)				χ²=68.4	<0.001
Normal Weight (<25)	308 (52.5)	252 (63.8)	56 (29.2)		
Overweight (25-29.9)	189 (32.2)	117 (29.6)	72 (37.5)		
Obese (≥30)	90 (15.3)	26 (6.6)	64 (33.3)		
Parity, n (%)				χ²=0.18	0.915
Nulliparous	321 (54.7)	217 (54.9)	104 (54.2)		
Multiparous	266 (45.3)	178 (45.1)	88 (45.8)		
Gravidity	2 (1, 3)	2 (1, 3)	2 (1, 3)	Z=-0.41	0.682
History of Prior GDM, n (%)	75 (12.8)	48 (12.2)	27 (14.1)	χ²=0.43	0.512
Family History of Diabetes, n (%)	163 (27.8)	107 (27.1)	56 (29.2)	χ²=0.29	0.589
Gestational Weight Gain up to GDM Dx (kg)	6.6 ± 3.3	6.5 ± 3.2	6.8 ± 3.5	t=1.05	0.294
Glycemic Markers at GDM Diagnosis					
FPG (mmol/L)	5.3 ± 0.6	5.1 ± 0.5	5.7 ± 0.6	t=12.5	<0.001
1hPG (mmol/L)	10.6 ± 1.4	10.3 ± 1.3	11.3 ± 1.4	t=8.56	<0.001
2hPG (mmol/L)	8.9 ± 1.2	8.7 ± 1.1	9.4 ± 1.3	t=7.05	<0.001
HbA1c (%)	5.5 ± 0.4	5.4 ± 0.3	5.7 ± 0.4	t=9.82	<0.001
Gestational age at outcome lipid assessment, weeks	34.4 (33.7, 35.2)	34.4 (33.7, 35.2)	34.5 (33.8, 35.3)	Z = −0.81	0.418
Outcome lipid assessment window, n (%)				χ² = 0.65	0.724
32 + 0 to 33 + 6 weeks	141 (24.0)	97 (24.6)	44 (22.9)		
34 + 0 to 35 + 6 weeks	339 (57.8)	226 (57.2)	113 (58.9)		
36 + 0 to 36 + 6 weeks	107 (18.2)	72 (18.2)	35 (18.2)		
First-Trimester Lipid Profile					
Triglycerides (mmol/L)	1.46 ± 0.53	1.35 ± 0.46	1.68 ± 0.60	t=7.12	<0.001
Total Cholesterol (mmol/L)	5.11 ± 0.86	5.05 ± 0.83	5.23 ± 0.91	t=2.35	0.019
HDL-C (mmol/L)	1.64 ± 0.39	1.69 ± 0.38	1.54 ± 0.39	t=-4.48	<0.001
LDL-C (mmol/L)	2.86 ± 0.73	2.77 ± 0.69	3.06 ± 0.77	t=4.51	<0.001
GDM Management, n (%)				χ²=46.1	<0.001
Lifestyle Only	408 (69.5)	309 (78.2)	99 (51.6)		
Metformin	131 (22.3)	72 (18.2)	59 (30.7)		
Insulin	48 (8.2)	14 (3.5)	34 (17.7)		

Data are presented as mean ± standard deviation, median (interquartile range), or n (%). BMI, Body Mass Index; GDM, Gestational Diabetes Mellitus; Dx, Diagnosis; FPG, Fasting Plasma Glucose; 1hPG, 1-hour Post-load Glucose; 2hPG, 2-hour Post-load Glucose; HbA1c, Glycated Hemoglobin; HDL-C, High-Density Lipoprotein Cholesterol; LDL-C, Low-Density Lipoprotein Cholesterol. Statistical tests used: Student’s t-test for normally distributed continuous variables (reported as t-value), Mann-Whitney U test for non-normally distributed continuous variables (reported as Z-value), and Chi-square test for categorical variables (reported as χ²-value).

### 3.2 LASSO regression variable selection and final model coefficients

**Figure 3:**
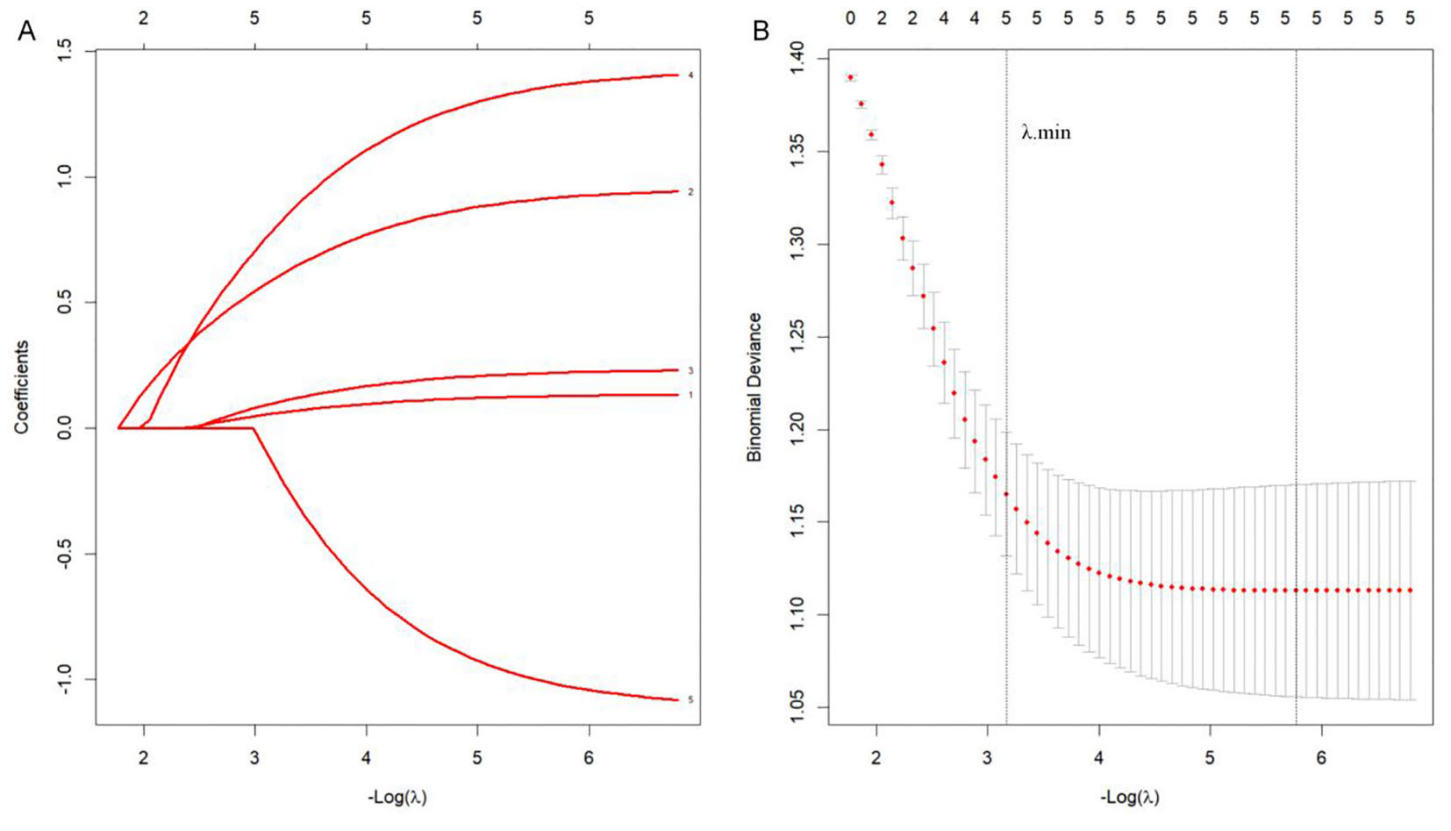
LASSO regression variable selection in the prediction of late-pregnancy hypertriglyceridemia. **(A)** Coefficient profiles of candidate predictors across a range of penalty parameters (log scale). Each red line represents the path of an individual predictor’s standardized coefficient as the regularization parameter λ varies. Variables included in the final model at the optimal λ minimizing binomial deviance are highlighted in red with labels adjacent to their coefficient curves. **(B)** Cross-validation curve displaying binomial deviance against log(λ) values. The vertical line indicates the optimal λ (λ.min) that yields the minimum cross-validated error. Dots represent mean cross-validated error; vertical bars represent one standard error.

The process of variable selection using LASSO logistic regression with 10-fold cross-validation on the training set (n=411) is visualized in **Figure 3A** (LASSO coefficient profiles) and **Figure 3B** (Cross-validation curve for tuning parameter selection). The optimal lambda (λ) value, lambda.min, which minimizes the binomial deviance, was identified and is clearly marked in **Figure 3B**. Using the prespecified selection-frequency rule across the 10 imputed training datasets, five predictors met the threshold for retention. Pre-gravid BMI, FPG at GDM diagnosis, and first-trimester TG were selected in 10/10 imputed datasets, 1hPG at GDM diagnosis was selected in 9/10 datasets, and first-trimester HDL-C was selected in 8/10 datasets. These predictors also showed the highest bootstrap inclusion frequencies, supporting their relative selection stability. In contrast, HbA1c, despite differing significantly between HTG and non-HTG groups in univariable comparisons, did not meet the selection threshold and had a lower bootstrap inclusion frequency, consistent with partial redundancy with OGTT-derived glucose markers. No candidate predictor showed severe multicollinearity, with all VIFs below 5. The complete candidate variable list, missingness rates, and VIFs are shown in **Supplementary Table 2**, and the LASSO selection frequency and bootstrap inclusion frequency of all candidate predictors are presented in **Supplementary Table 3**. These five predictors were then used to construct a standard multivariable logistic regression model on the training set to obtain the final, unpenalized coefficients for clinical application. The results of this final model are presented in **Table 2**. All five variables were independently and significantly associated with the risk of developing late-pregnancy HTG. Higher pre-gravid BMI, FPG, 1hPG, and first-trimester TG levels were associated with an increased risk, while higher first-trimester HDL-C was associated with a decreased risk.

**Table 2:** Final multivariable logistic regression model for predicting late-pregnancy hypertriglyceridemia.

Predictor	β coefficient	Standard error (SE)	Adjusted odds ratio (OR)	95% CI for OR	P-value
Pre-gravid BMI (per kg/m²)	0.138	0.027	1.15	1.09 – 1.21	<0.001
FPG at GDM Diagnosis (per mmol/L)	0.921	0.184	2.51	1.75 – 3.60	<0.001
1hPG at GDM Diagnosis (per mmol/L)	0.283	0.081	1.33	1.13 – 1.56	<0.001
First-Trimester TG (per mmol/L)	0.655	0.198	1.92	1.31 – 2.84	0.001
First-Trimester HDL-C (per mmol/L)	-0.892	0.301	0.41	0.23 – 0.74	0.003
Intercept	-10.215	1.842	–	–	<0.001

The final model was developed on the training set (n=411) using the predictors selected by the LASSO regression procedure. The model is presented as: Log-odds (HTG) = -10.215 + 0.138*(Pre-gravid BMI) + 0.921*(FPG) + 0.283*(1hPG) + 0.655*(First-Trimester TG) - 0.892*(First-Trimester HDL-C). BMI, Body Mass Index; FPG, Fasting Plasma Glucose; GDM, Gestational Diabetes Mellitus; 1hPG, 1-hour Post-load Glucose; TG, Triglycerides; HDL-C, High-Density Lipoprotein Cholesterol; OR, Odds Ratio; CI, Confidence Interval.

### 3.3 Comparison of training and testing sets

To assess the balance of the 7:3 split into training and held-out internal test sets, the baseline characteristics of the two cohorts were compared. As detailed in **Table 3**, no statistically significant differences were observed between the training set (n=411) and the testing set (n=176) across all demographic, clinical, and laboratory variables examined (all P > 0.05). This indicated that the random allocation produced two comparable subsets, allowing a secondary held-out assessment of model performance within the same source population. However, because this split reduced the sample available for model development, bootstrap optimism correction using the full analytic cohort was prespecified as the primary internal validation approach.

**Table 3:** Comparison of baseline characteristics between the training and testing sets.

Characteristic	Training set (n=411)	Testing set (n=176)	Statistical value	P-value
Maternal Age (years)	31.3 ± 4.9	30.9 ± 4.9	t=0.92	0.357
Pre-gravid BMI (kg/m²)	25.4 ± 4.8	25.1 ± 4.7	t=0.67	0.505
Pre-gravid BMI Category, n (%)			χ²=0.61	0.737
Normal Weight (<25)	213 (51.8)	95 (54.0)		
Overweight (25-29.9)	135 (32.8)	54 (30.7)		
Obese (≥30)	63 (15.3)	27 (15.3)		
Parity, n (%)			χ²=0.04	0.850
Nulliparous	223 (54.3)	98 (55.7)		
Multiparous	188 (45.7)	78 (44.3)		
Gravidity	2 (1, 3)	2 (1, 3)	Z=-0.29	0.775
History of Prior GDM, n (%)	51 (12.4)	24 (13.6)	χ²=0.17	0.684
Family History of Diabetes, n (%)	116 (28.2)	47 (26.7)	χ²=0.14	0.705
Gestational Weight Gain up to GDM Dx (kg)	6.5 ± 3.3	6.7 ± 3.2	t=-0.68	0.495
Glycemic Markers at GDM Diagnosis				
FPG (mmol/L)	5.3 ± 0.6	5.3 ± 0.6	t=0.15	0.883
1hPG (mmol/L)	10.6 ± 1.4	10.6 ± 1.4	t=0.28	0.777
2hPG (mmol/L)	8.9 ± 1.2	8.9 ± 1.2	t=0.10	0.923
HbA1c (%)	5.5 ± 0.4	5.5 ± 0.4	t=0.63	0.531
First-Trimester Lipid Profile				
Triglycerides (mmol/L)	1.46 ± 0.53	1.45 ± 0.52	t=0.22	0.829
Total Cholesterol (mmol/L)	5.12 ± 0.86	5.09 ± 0.85	t=0.40	0.692
HDL-C (mmol/L)	1.64 ± 0.39	1.65 ± 0.38	t=-0.30	0.762
LDL-C (mmol/L)	2.87 ± 0.73	2.84 ± 0.72	t=0.47	0.641
GDM Management, n (%)			χ²=1.84	0.399
Lifestyle Only	292 (71.0)	116 (65.9)		
Metformin	89 (21.7)	42 (23.9)		
Insulin	30 (7.3)	18 (10.2)		
Late-Pregnancy HTG, n (%)	134 (32.6)	58 (33.0)	χ²=0.01	0.928

Data are presented as mean ± standard deviation, median (interquartile range), or n (%). BMI, Body Mass Index; GDM, Gestational Diabetes Mellitus; Dx, Diagnosis; FPG, Fasting Plasma Glucose; 1hPG, 1-hour Post-load Glucose; 2hPG, 2-hour Post-load Glucose; HbA1c, Glycated Hemoglobin; HDL-C, High-Density Lipoprotein Cholesterol; LDL-C, Low-Density Lipoprotein Cholesterol; HTG, Hypertriglyceridemia. Statistical tests used: Student’s t-test for normally distributed continuous variables (reported as t-value), Mann-Whitney U test for non-normally distributed continuous variables (reported as Z-value), and Chi-square test for categorical variables (reported as χ²-value).

### 3.4 Model performance evaluation

The performance of the final prediction model was evaluated in terms of discrimination, calibration, overall prediction error, bootstrap optimism correction, and threshold-specific classification metrics (**Table 4**). In the held-out internal test set (n = 176), the model showed good discrimination, with a C-statistic (AUC) of 0.816 (95% CI: 0.754–0.878), as shown in **Figure 1A**. Calibration was acceptable, with calibration-in-the-large of −0.03, calibration slope of 0.94, and no significant lack of fit on the Hosmer-Lemeshow test (χ² = 7.24, P = 0.510). The Brier score was 0.151, and the scaled Brier score was 0.301. Decile-level calibration showed that observed event proportions generally followed the mean predicted probabilities across the risk spectrum, with modest deviations in several middle-risk groups, as detailed in **Supplementary Table 4**. The apparent and bootstrap bias-corrected calibration curves are presented in **Figure 1B**.

**Table 4:** Performance of the final prediction model and classification metrics at clinically meaningful thresholds.

Metric	Value
Overall model performance in the held-out internal test set	
AUC/C-statistic	0.816
95% CI for AUC	0.754–0.878
Calibration-in-the-large	−0.03
Calibration slope	0.94
Hosmer-Lemeshow P value	0.510
Brier score	0.151
Scaled Brier score	0.301
Bootstrap optimism-corrected performance	
Optimism-corrected AUC/C-statistic	0.805
Optimism-corrected calibration-in-the-large	−0.02
Optimism-corrected calibration slope	0.91
Optimism-corrected Brier score	0.158
Optimism-corrected scaled Brier score	0.269
Classification performance at clinically meaningful thresholds	
Threshold 0.10: sensitivity	98.3%
Threshold 0.10: specificity	32.2%
Threshold 0.10: PPV	42.6%
Threshold 0.10: NPV	96.9%
Threshold 0.20: sensitivity	91.4%
Threshold 0.20: specificity	61.9%
Threshold 0.20: PPV	53.5%
Threshold 0.20: NPV	91.7%
Threshold 0.30: sensitivity	82.8%
Threshold 0.30: specificity	72.9%
Threshold 0.30: PPV	60.8%
Threshold 0.30: NPV	88.9%
Threshold 0.34, Youden-J optimum: sensitivity	75.4%
Threshold 0.34, Youden-J optimum: specificity	75.8%
Threshold 0.34, Youden-J optimum: PPV	62.7%
Threshold 0.34, Youden-J optimum: NPV	85.2%
Threshold 0.40: sensitivity	65.5%
Threshold 0.40: specificity	84.7%
Threshold 0.40: PPV	69.2%
Threshold 0.40: NPV	82.3%
Threshold 0.50: sensitivity	46.6%
Threshold 0.50: specificity	93.2%
Threshold 0.50: PPV	77.1%
Threshold 0.50: NPV	77.3%

Overall model performance was evaluated in the held-out internal test set, and optimism-corrected estimates were obtained using 1000 bootstrap resamples of the full analytic cohort. Classification metrics were calculated in the held-out internal test set at prespecified clinically meaningful risk thresholds. The 0.34 threshold corresponds to the Youden-J optimum used for the primary classification analysis. Lower thresholds favor sensitivity and negative predictive value, whereas higher thresholds favor specificity and positive predictive value. AUC, area under the receiver operating characteristic curve; CI, confidence interval; PPV, positive predictive value; NPV, negative predictive value.

Bootstrap validation using 1000 resamples of the full analytic cohort showed limited optimism. The optimism-corrected C-statistic was 0.805, corresponding to an optimism estimate of 0.011. The optimism-corrected calibration-in-the-large was −0.02, and the optimism-corrected calibration slope was 0.91. The optimism-corrected Brier score and scaled Brier score were 0.158 and 0.269, respectively, indicating acceptable internal performance after correction for optimism.

Classification performance varied across clinically meaningful thresholds. As expected, lower thresholds favored sensitivity and NPV, whereas higher thresholds improved specificity and PPV. At a 0.20 threshold, sensitivity was 91.4% and NPV was 91.7%, supporting a rule-out-oriented strategy. At a 0.40 threshold, specificity increased to 84.7% and PPV to 69.2%, supporting a more selective high-risk monitoring strategy. At the Youden-J optimum threshold of 0.34, sensitivity, specificity, PPV, NPV, and accuracy were 75.4%, 75.8%, 62.7%, 85.2%, and 75.6%, respectively. Full threshold-specific classification metrics are presented in **Table 4**.

The full five-variable model showed better discrimination and overall prediction accuracy than the reference models. In the held-out internal test set, the AUC was 0.681 (95% CI: 0.604–0.758) for first-trimester TG alone, 0.735 (95% CI: 0.666–0.804) for pre-gravid BMI plus first-trimester TG, and 0.784 (95% CI: 0.716–0.852) for the four-variable model excluding first-trimester TG. Compared with the four-variable model, the full five-variable model improved the AUC by 0.032 (P = 0.041), reduced the Brier score from 0.164 to 0.151, and increased the scaled Brier score from 0.241 to 0.301. Decision curve analysis further showed that the full model provided a modestly higher net benefit than the four-variable model across threshold probabilities of approximately 0.20–0.45. Detailed model comparisons are presented in **Supplementary Table 5**.

### 3.5 Subgroup analysis of model performance

**Figure 4:**
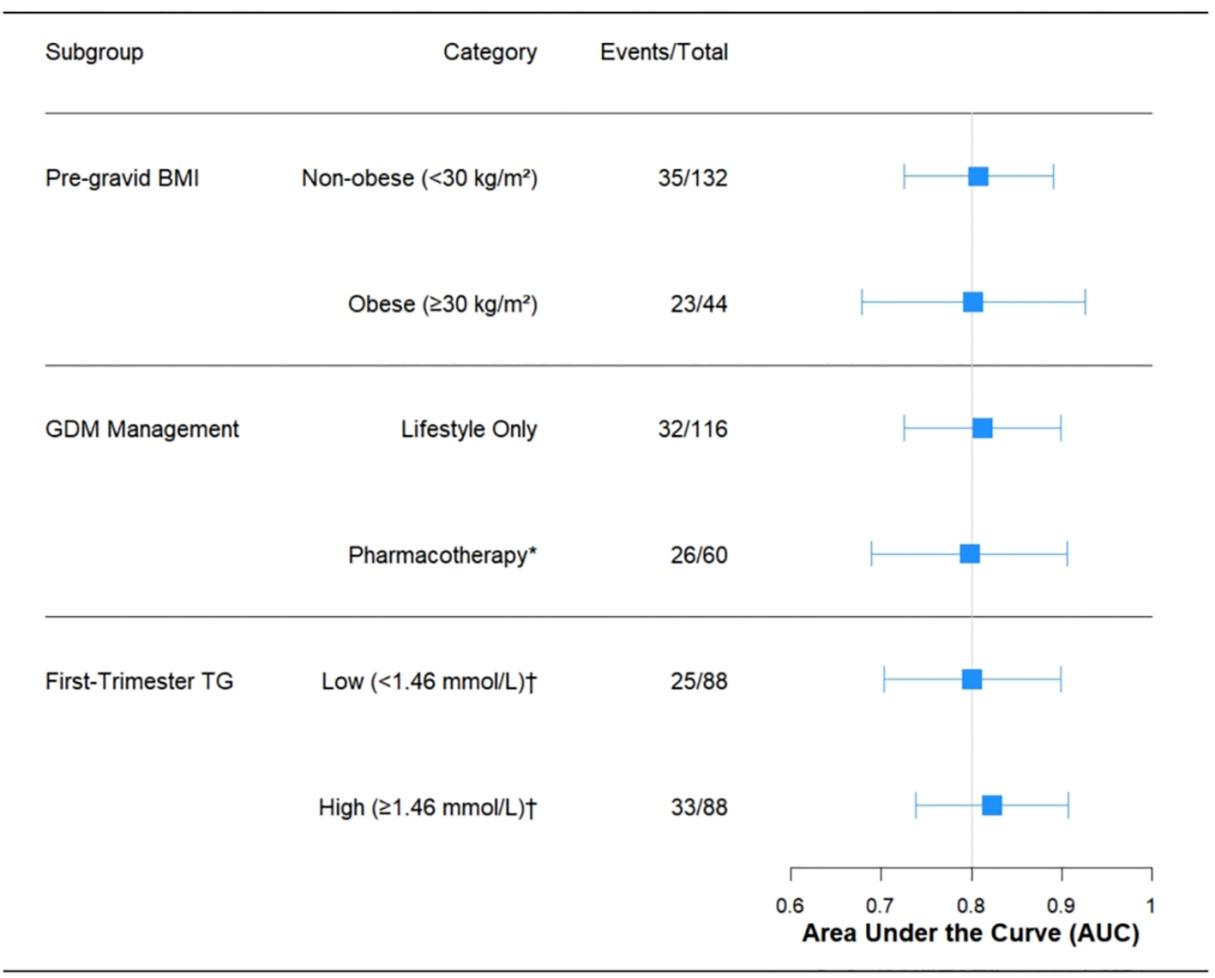
Forest plot illustrating the model’s predictive discrimination (area under the curve, AUC) across clinically relevant patient subgroups. Each row corresponds to a subgroup category, with the left panel showing subgroup name, category, and “events/total” counts. The right panel visualizes the model’s AUC point estimates (blue squares) with 95% confidence intervals (horizontal lines). The vertical reference line at 0.8 represents a key discrimination threshold. Overlapping CIs indicate no significant difference in model performance across subgroups.

To explore the consistency of model discrimination across clinically relevant patient subgroups, we evaluated its discriminatory performance (AUC) in the test set within predefined strata (**Figure 4**). The model maintained consistently strong performance across all subgroups, with no statistically significant interaction observed between the subgroup variables and the model’s predictive ability (all P for interaction > 0.05). Specifically, the AUC remained high and comparable between women who were non-obese vs. obese (0.808 vs. 0.802), between those managed by lifestyle alone vs. those requiring pharmacotherapy (0.812 vs. 0.798), and between those with low vs. high first-trimester TG levels (0.801 vs. 0.823). These exploratory subgroup findings suggested broadly consistent discrimination across the assessed subgroups, although they should be interpreted cautiously because of limited subgroup sample sizes.

### 3.6 Model visualization and clinical application

**Figure 5:**
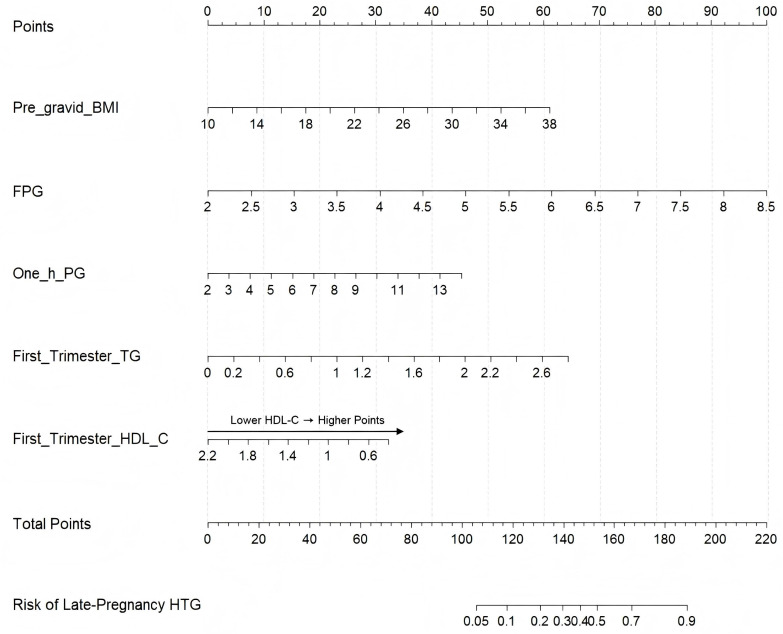
Nomogram for predicting the risk of late-pregnancy hypertriglyceridemia (HTG) in women with gestational diabetes mellitus (GDM). For each predictor, a vertical line is drawn upward to the “Points” axis to determine the corresponding score, and the total score is then mapped to the predicted risk of late-pregnancy HTG. For the First-Trimester HDL-C axis, lower HDL-C values correspond to higher point assignments, as indicated by the directional annotation.

**Figure 6:**
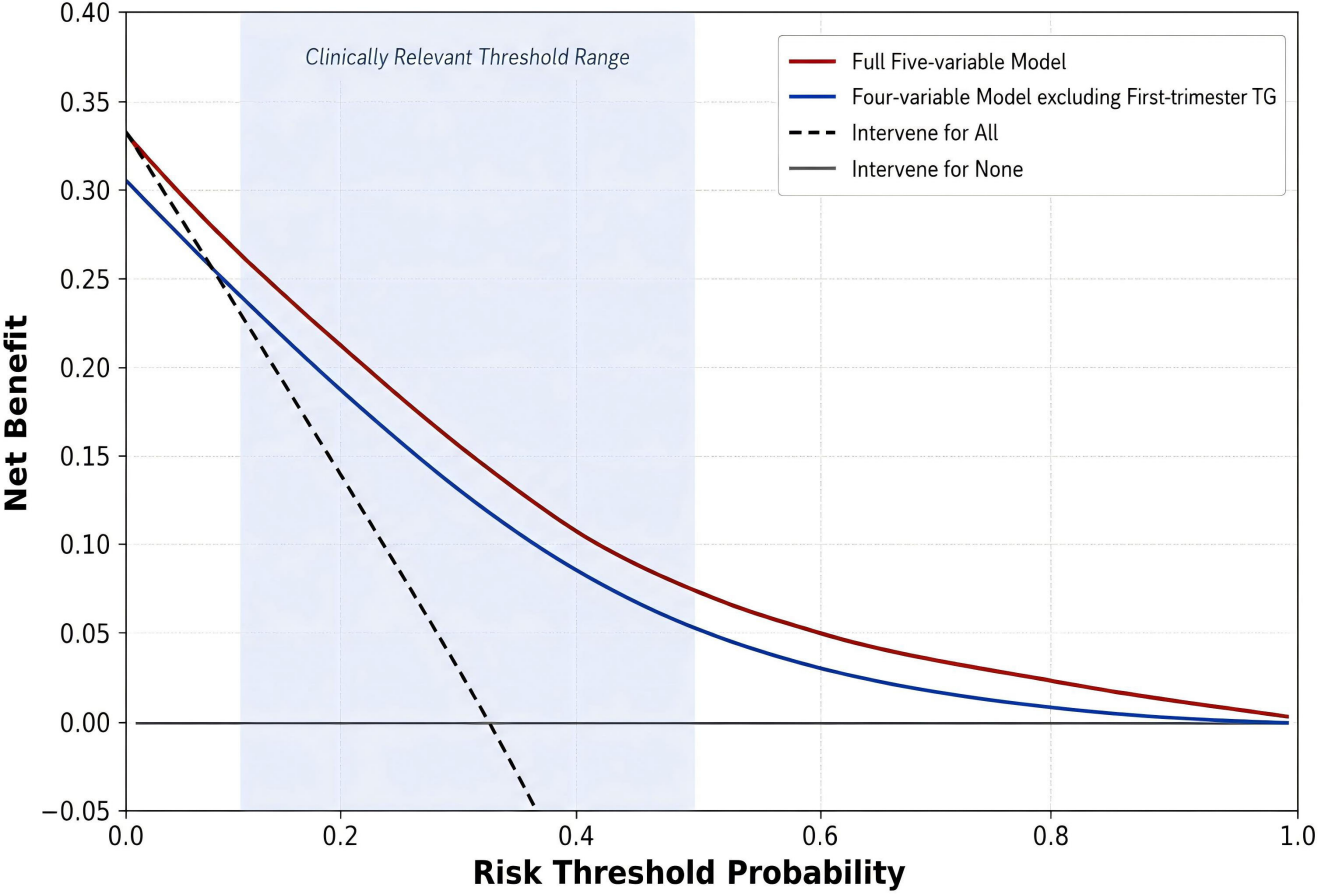
Decision curve analysis comparing the clinical utility of the full five-variable model and the four-variable model excluding first-trimester TG in the held-out internal test set. The red solid line represents the full five-variable model, and the blue solid line represents the four-variable model excluding first-trimester TG. The black dashed line represents the strategy of intervening for all patients, and the gray solid line represents the strategy of intervening for no patients. The shaded area indicates the prespecified clinically relevant threshold-probability range of 0.10 to 0.50. Within this range, the full model showed a modestly higher net benefit than the four-variable model, particularly at threshold probabilities of approximately 0.20–0.45.

To facilitate the clinical application of the prediction model, a nomogram was constructed based on the final multivariable logistic regression coefficients (**Figure 5**). This graphical tool allows clinicians to easily calculate an individualized probability of late-pregnancy HTG for a patient by summing the points assigned to each of the five predictor values (pre-gravid BMI, FPG, 1hPG, first-trimester TG, and first-trimester HDL-C) and projecting the total points to the bottom risk scale. The clinical utility of the model was quantitatively assessed using DCA in the test set across prespecified threshold probabilities ranging from 0.10 to 0.50. As presented in **Figure 6**, the DCA demonstrated that employing the prediction model to guide decisions on intensified monitoring or intervention provided a superior net benefit compared with the default strategies of intervening for all or intervening for none across the majority of threshold probabilities. In the incremental analysis, the full five-variable model showed a modestly higher net benefit than the four-variable model excluding first-trimester TG, particularly at threshold probabilities of approximately 0.20–0.45. This indicates that using the model in clinical practice would lead to better patient outcomes by correctly identifying high-risk individuals for targeted management while avoiding unnecessary interventions in low-risk women.

The relationship between the total points and the predicted probability follows the standard logistic transformation: Risk = 1/[1 + exp −(α + β × Total Points)], where α and β are the intercept and scaling coefficients derived from the final logistic regression model. The total points correspond linearly to the log-odds of late-pregnancy HTG, and the bottom axis of the nomogram converts this linear predictor into an individualized predicted probability.

### 3.7 Sensitivity analyses

In the first sensitivity analysis, gestational age at outcome lipid measurement was additionally included in the final model to account for residual variation in measurement timing within the predefined 32 + 0 to 36 + 6-week window. The coefficient for gestational age at lipid measurement was small and not statistically significant (OR = 1.06 per week, 95% CI: 0.89–1.27, P = 0.502). Model discrimination remained essentially unchanged in the held-out internal test set, with an AUC of 0.814 (95% CI: 0.752–0.876), compared with 0.816 (95% CI: 0.754–0.878) in the primary model. Calibration also remained adequate, with a Hosmer-Lemeshow test P value of 0.487. The PPV and NPV were 62.1% and 84.9%, respectively. These findings suggested that residual measurement-time variation within the predefined outcome window did not materially affect model performance. The detailed comparison between the primary model and the sensitivity model additionally adjusted for gestational age at lipid measurement is presented in **Supplementary Table 6**.

In the second set of sensitivity analyses, alternative TG cut-offs were applied within the same predefined 32 + 0 to 36 + 6-week outcome window to evaluate whether model performance was sensitive to the outcome threshold. The incidence of HTG varied as expected across thresholds: 70.0% (411/587) using ≥1.7 mmol/L, 32.7% (192/587) using the primary ≥2.3 mmol/L threshold, 25.9% (152/587) using ≥2.5 mmol/L, and 16.4% (96/587) using ≥2.83 mmol/L. Model discrimination in the held-out internal test set remained acceptable across these definitions, with AUC values of 0.781, 0.816, 0.807, and 0.789 for the ≥1.7, ≥2.3, ≥2.5, and ≥2.83 mmol/L thresholds, respectively. Calibration remained adequate across the alternative definitions. As expected, PPV and NPV varied with the outcome prevalence across thresholds, whereas the overall predictor structure remained directionally consistent. The detailed results are presented in **Supplementary Table 7**.

In the sensitivity analysis replacing self-reported pre-pregnancy BMI with BMI calculated from first measured antenatal weight, model performance remained materially unchanged. The AUC in the held-out internal test set was 0.811 (95% CI: 0.748–0.874), compared with 0.816 (95% CI: 0.754–0.878) in the primary model. Calibration remained acceptable, with calibration-in-the-large of −0.04, calibration slope of 0.92, and Hosmer-Lemeshow test P = 0.462. The Brier score was 0.154. At the same probability threshold, sensitivity, specificity, PPV, and NPV were 74.1%, 75.0%, 61.8%, and 84.6%, respectively (**Supplementary Table 8**). These findings suggested that the model was not materially affected by the use of self-reported pre-pregnancy weight.

## 4 Discussion

In this study, we successfully developed and validated a predictive nomogram for late-gestational HTG in patients with GDM, utilizing five readily available clinical indicators at the time of GDM diagnosis. The model demonstrated good discrimination, acceptable calibration, and clinical utility in internal validation. This research addresses a critical gap by providing an early-warning tool that enables risk stratification, potentially guiding personalized monitoring and intervention strategies to mitigate the adverse metabolic consequences associated with severe HTG in the context of GDM. Our findings represent a significant step toward proactive and individualized management of dyslipidemia in this high-risk obstetric population.

The five predictors incorporated into our final model are: pre-pregnancy BMI, early-gestation TG, HDL-C, FPG, and 1hPG. These variables are well-supported by existing pathophysiological evidence, as pre-pregnancy BMI and early-gestation lipid levels serve as markers of the patient’s baseline metabolic state. Obesity and preexisting dyslipidemia are foundational to the insulin resistance that characterizes GDM, setting the stage for subsequent metabolic decompensation (16). Concurrently, FPG and 1hPG are direct indicators of the severity of glucose metabolism disruption at the time of GDM diagnosis. FPG primarily reflects hepatic insulin resistance, while the 1hPG level is a strong indicator of both β-cell function and peripheral insulin sensitivity (17). Together, these glucose metrics capture the intensity of the metabolic stress that drives further lipid dysregulation (18). While previous studies have identified associations between individual factors like BMI, early-pregnancy TG, and GDM-related adverse outcomes (19, 20), our work is novel in its integration of these variables into a quantitative, multivariable predictive model specifically designed for late-gestational HTG risk in the GDM population. This integrated approach provides a more comprehensive and personalized risk assessment than relying on single risk factors or arbitrary cutoffs (21). Because first-trimester TG and late-pregnancy HTG represent measurements of the same lipid component at different gestational stages, we acknowledge that inclusion of first-trimester TG may partly increase discrimination through temporal autocorrelation. However, the four-variable model excluding first-trimester TG still showed acceptable performance, whereas adding first-trimester TG further improved the AUC from 0.784 to 0.816 and improved the Brier score from 0.164 to 0.151. This incremental gain was modest but clinically relevant, particularly because first-trimester lipid testing is routinely available in many antenatal care settings and does not require an additional specialized test when already included in early pregnancy metabolic assessment.

Our model demonstrated good discrimination, with an AUC of approximately 0.81. A particularly valuable feature is its high NPV of 85.2%, suggesting that the model can reliably identify a large subgroup of GDM patients at low risk for developing late-pregnancy HTG. This capability could be instrumental in optimizing healthcare resource allocation by potentially reducing the intensity of lipid monitoring for these low-risk individuals. The development of a nomogram translates the statistical model into a user-friendly, graphical tool for point-of-care risk estimation. Furthermore, our decision curve analysis (DCA) quantitatively confirmed the model’s clinical utility. The DCA showed that using our model to guide clinical decisions provides a greater net benefit across a wide range of clinically relevant risk thresholds compared to the default strategies of treating all or no patients. The consistency of the model’s performance across subgroups defined by BMI, baseline lipid levels, and treatment modalities further underscores its robustness and generalizability within our study population. The use of DCA is critical for demonstrating that a statistically significant model is also clinically valuable and can lead to better patient outcomes (22).

This study possesses several notable strengths. First, its development and reporting adhere to the TRIPOD guidelines, ensuring clarity and reproducibility (23). Second, the temporal design, using only predictors available at or before GDM diagnosis to predict a future event, strengthens the potential for causal inference. Third, we used complementary internal performance assessments. The held-out test set provided a transparent secondary assessment within the same source population, whereas bootstrap optimism correction using the full analytic cohort was treated as the primary internal validation approach because it better preserves sample information and estimates optimism in discrimination, calibration, and overall prediction error. Finally, our focus extends beyond statistical performance to clinical applicability, as evidenced by the inclusion of the nomogram and DCA. Beyond regression-based prediction tools, recent studies have also explored machine-learning approaches for GDM risk stratification. For example, Xing et al. developed GDMPredictor, a machine-learning-based web server using clinical and biochemical indicators to predict GDM risk and support individualized management (24). Such approaches may complement parsimonious nomogram-based tools, particularly when larger, multicenter datasets and high-dimensional biochemical or lifestyle variables are available. However, the target of the present model differs from these GDM-onset prediction tools, as our study focused on predicting late-pregnancy HTG among women already diagnosed with GDM. Future research should compare traditional regression-based nomograms with machine-learning models for this specific outcome and evaluate whether more complex algorithms provide clinically meaningful incremental benefit.

However, some limitations must be acknowledged. The single-center, retrospective design is inherently susceptible to selection bias and potential inaccuracies in recorded data. Pre-pregnancy weight was mainly self-reported, which may have introduced measurement error in pre-gravid BMI. However, agreement with the first measured antenatal weight was high, and replacing self-reported pre-pregnancy BMI with BMI derived from first measured antenatal weight produced similar discrimination, calibration, and overall prediction accuracy. Nevertheless, future prospective studies should use standardized measured pre-pregnancy or early-pregnancy weight whenever feasible. Another limitation is that post-diagnosis management factors were not incorporated into the prediction model. Because the model was designed for risk estimation at the time of GDM diagnosis, subsequent lifestyle intervention, metformin or insulin therapy, and later gestational weight gain occurred after the prediction time point and were therefore not used as predictors. However, these time-varying factors may modify late-pregnancy lipid trajectories and affect the model’s real-world predictive performance, particularly its positive predictive value. Future prospective studies should evaluate whether dynamic updating with treatment response and gestational weight gain improves risk prediction. In addition, patients without a qualifying lipid measurement within the predefined 32 + 0 to 36 + 6-week outcome window were excluded, including those whose outcome window extended beyond the data collection cut-off date. This approach ensured clear outcome classification but may have introduced selection bias if excluded women differed systematically from the analytic cohort. The predefined 32 + 0 to 36 + 6-week outcome window was used to reduce measurement-time heterogeneity compared with an unrestricted last-available lipid measurement before delivery. In the sensitivity analysis, additional adjustment for gestational age at lipid measurement did not materially change model performance, suggesting that residual timing variation within this window was unlikely to drive the main findings. The subgroup analyses should also be interpreted cautiously because several subgroups had small sample sizes, which limited statistical power and increased the uncertainty of subgroup-specific AUC estimates. Although we used multiple imputation to handle missing values, this process can introduce a degree of uncertainty. Another limitation is that no universally accepted pregnancy-specific TG threshold exists for defining clinically relevant late-pregnancy HTG. We therefore used TG ≥ 2.3 mmol/L as a pragmatic primary threshold for moderate late-pregnancy TG elevation and performed sensitivity analyses using alternative cut-offs of ≥1.7, ≥2.5, and ≥2.83 mmol/L. The incidence of HTG varied across thresholds, but model discrimination remained acceptable, supporting the robustness of the predictor structure while underscoring that absolute incidence and predictive values are threshold-dependent. A primary limitation is the absence of an independent external validation cohort. Although split-sample testing and bootstrap resampling were used for internal validation, these procedures cannot replace validation in a separate population. Therefore, the current model should not yet be considered broadly generalizable, and its applicability is presently restricted to GDM populations with baseline characteristics, clinical management patterns, and outcome-assessment practices similar to those of the present single-center cohort. In addition, because women with chronic hypertension, cardiovascular disease, thyroid dysfunction, or severe hepatic or renal insufficiency were excluded to reduce confounding from pre-existing conditions that could substantially influence lipid metabolism, the study population should be regarded as a selected GDM cohort, and applicability to higher-risk GDM patients with major metabolic, endocrine, cardiovascular, or organ-system comorbidities remains uncertain. We also acknowledge that other potential predictors, such as detailed dietary information, physical activity levels, or novel biomarkers, were not available for inclusion. Prospective external validation in multicenter and geographically diverse cohorts is required before routine clinical implementation can be recommended (25).

## 5 Conclusion

This study presents an internally validated, practical tool for the early prediction of late-gestational hypertriglyceridemia in women with GDM. The model, based on five routinely collected clinical parameters, may support individualized risk stratification at the time of GDM diagnosis in populations similar to the derivation cohort, particularly where first-trimester lipid testing is already part of routine antenatal metabolic assessment. This allows for the implementation of a targeted management strategy, where high-risk individuals could receive intensified lipid monitoring and proactive interventions, such as enhanced dietary counseling and lifestyle modifications, while low-risk individuals might follow a less intensive management pathway. Looking forward, the essential next step is the prospective external validation of this model in diverse populations. Subsequent research should focus on evaluating whether risk stratification based on this tool, followed by targeted interventions, can effectively reduce the incidence of severe HTG and improve maternal and neonatal outcomes. The long-term consequences of GDM-associated HTG on both the mother and offspring, including the heightened risk of future cardiovascular disease, underscore the importance of such preventative strategies. Investigating the integration of additional biomarkers or lifestyle factors may further refine the model’s predictive accuracy and clinical impact.

## Data Availability

The original contributions presented in the study are included in the article/**Supplementary Material.** Further inquiries can be directed to the corresponding author.
