# Supplementary material for: Early prediction of late-pregnancy hypertriglyceridemia in women with gestational diabetes: development and internal validation of a clinical risk model
Source: Front Endocrinol (Lausanne). 2026 Jun 26;17:1845573. doi: 10.3389/fendo.2026.1845573 (PMC13350170; doi:10.3389/fendo.2026.1845573)
Supplement: Supplementary file 1 [file Table1.docx]

**Supplementary Table S1.** Agreement between self-reported pre-pregnancy weight and first measured antenatal weight

| **Metric** | **Value** |
| --- | --- |
| Women with first measured antenatal weight available | 552/587 (94.0%) |
| Gestational age at first measured antenatal weight, weeks | 9.6 (8.4–11.2) |
| Self-reported pre-pregnancy weight, kg | 65.1 ± 11.8 |
| First measured antenatal weight, kg | 65.9 ± 11.9 |
| Mean difference, kg | −0.8 ± 2.4 |
| Pearson correlation coefficient | 0.97 |
| ICC | 0.96 |
| 95% CI for ICC | 0.95–0.97 |
| Bland–Altman 95% limits of agreement, kg | −5.5 to 3.9 |
| BMI category agreement | 91.8% |
| Weighted κ for BMI category | 0.87 |

Notes: Mean difference was calculated as self-reported pre-pregnancy weight minus first measured antenatal weight. Negative values indicate lower self-reported weight. BMI category agreement was assessed using WHO BMI categories. ICC, intraclass correlation coefficient; BMI, body mass index.

**Supplementary Table S2.** Candidate predictors, missingness, and multicollinearity assessment before model selection

| **Candidate predictor** | **Category** | **Missing values in final analytic cohort (N = 587), n (%)** | **VIF** | **Included in LASSO candidate set** |
| --- | --- | --- | --- | --- |
| Maternal age | Demographic | 0 (0.0) | 1.21 | Yes |
| Pre-gravid BMI | Demographic/metabolic | 0 (0.0) | 1.42 | Yes |
| Parity | Obstetric history | 0 (0.0) | 2.63 | Yes |
| Gravidity | Obstetric history | 0 (0.0) | 2.71 | Yes |
| History of prior GDM | Obstetric history | 7 (1.2) | 1.18 | Yes |
| Family history of diabetes | Family history | 18 (3.1) | 1.16 | Yes |
| Gestational weight gain up to GDM diagnosis | Pregnancy weight trajectory | 21 (3.6) | 1.31 | Yes |
| FPG at GDM diagnosis | OGTT-derived glycemic marker | 0 (0.0) | 2.18 | Yes |
| 1hPG at GDM diagnosis | OGTT-derived glycemic marker | 0 (0.0) | 1.96 | Yes |
| 2hPG at GDM diagnosis | OGTT-derived glycemic marker | 0 (0.0) | 1.88 | Yes |
| HbA1c at GDM diagnosis | Glycemic marker | 46 (7.8) | 2.45 | Yes |
| First-trimester TG | First-trimester lipid marker | 0 (0.0) | 1.92 | Yes |
| First-trimester TC | First-trimester lipid marker | 0 (0.0) | 2.74 | Yes |
| First-trimester HDL-C | First-trimester lipid marker | 0 (0.0) | 1.48 | Yes |
| First-trimester LDL-C | First-trimester lipid marker | 0 (0.0) | 2.56 | Yes |
| GDM management strategy | Post-diagnosis management variable | 0 (0.0) | Not assessed | No |

Notes: Candidate predictors were defined as variables available at or before GDM diagnosis and considered for LASSO-based model selection. GDM management strategy was collected descriptively but was not entered into LASSO because it occurred after the prediction time point. VIFs were calculated among candidate predictors before penalized regression to assess multicollinearity. All VIFs were below 5, indicating no severe multicollinearity. Missing values were handled using multiple imputation by chained equations. Abbreviations: BMI, body mass index; GDM, gestational diabetes mellitus; VIF, variance inflation factor; LASSO, least absolute shrinkage and selection operator; FPG, fasting plasma glucose; 1hPG, 1-hour post-load glucose; 2hPG, 2-hour post-load glucose; HbA1c, glycated hemoglobin; OGTT, oral glucose tolerance test; TG, triglycerides; TC, total cholesterol; HDL-C, high-density lipoprotein cholesterol; LDL-C, low-density lipoprotein cholesterol.

**Supplementary Table S3.** LASSO selection frequency and bootstrap inclusion frequency of candidate predictors

| **Candidate predictor** | **Selection frequency across 10 imputed training datasets, n/10** | **Bootstrap inclusion frequency, %** | **Retained in final model** |
| --- | --- | --- | --- |
| First-trimester TG | 10/10 | 91.3 | Yes |
| Pre-gravid BMI | 10/10 | 86.7 | Yes |
| FPG at GDM diagnosis | 10/10 | 82.4 | Yes |
| 1hPG at GDM diagnosis | 9/10 | 76.1 | Yes |
| First-trimester HDL-C | 8/10 | 69.5 | Yes |
| HbA1c at GDM diagnosis | 4/10 | 38.2 | No |
| 2hPG at GDM diagnosis | 3/10 | 34.6 | No |
| First-trimester LDL-C | 3/10 | 31.4 | No |
| First-trimester TC | 2/10 | 27.8 | No |
| Gestational weight gain up to GDM diagnosis | 2/10 | 22.6 | No |
| Maternal age | 2/10 | 20.9 | No |
| Family history of diabetes | 1/10 | 16.3 | No |
| Parity | 1/10 | 13.1 | No |
| Gravidity | 0/10 | 10.7 | No |
| History of prior GDM | 0/10 | 8.5 | No |

Notes: LASSO logistic regression with 10-fold cross-validation was performed independently in each of the 10 imputed training datasets. In each imputed dataset, the optimal penalty parameter was selected using lambda.min, and predictors with non-zero coefficients at this penalty were recorded as selected. A prespecified selection-frequency rule was applied, whereby predictors selected in at least 6 of the 10 imputed datasets were retained for the final multivariable logistic regression model. Bootstrap inclusion frequency was calculated from 1000 patient-level bootstrap replicates, with the full modeling workflow repeated in each replicate. Abbreviations: LASSO, least absolute shrinkage and selection operator; GDM, gestational diabetes mellitus; FPG, fasting plasma glucose; 1hPG, 1-hour post-load glucose; 2hPG, 2-hour post-load glucose; TG, triglycerides; TC, total cholesterol; HDL-C, high-density lipoprotein cholesterol; LDL-C, low-density lipoprotein cholesterol; HbA1c, glycated hemoglobin.

**Supplementary Table S4.** Decile-level calibration in the held-out internal test set

| **Risk decile** | **No. of patients** | **Mean predicted probability** | **Observed HTG events, n** | **Observed probability** |
| --- | --- | --- | --- | --- |
| 1 | 18 | 0.06 | 1 | 0.06 |
| 2 | 18 | 0.10 | 2 | 0.11 |
| 3 | 18 | 0.15 | 3 | 0.17 |
| 4 | 18 | 0.21 | 3 | 0.17 |
| 5 | 18 | 0.27 | 5 | 0.28 |
| 6 | 18 | 0.33 | 6 | 0.33 |
| 7 | 17 | 0.40 | 7 | 0.41 |
| 8 | 17 | 0.49 | 8 | 0.47 |
| 9 | 17 | 0.60 | 10 | 0.59 |
| 10 | 17 | 0.72 | 13 | 0.76 |

**Notes:** Patients in the held-out internal test set were grouped into deciles according to predicted probability. The observed probability was calculated as the number of late-pregnancy HTG events divided by the number of patients in each decile. The table shows modest decile-level variation around the mean predicted probabilities, supporting acceptable but not perfect calibration. Abbreviations: HTG, hypertriglyceridemia.

**Supplementary Table S5.** Comparison of reference models and incremental predictive value of first-trimester TG

| **Metric** | **First-trimester TG alone** | **BMI + first-trimester TG** | **Four-variable model excluding first-trimester TG** | **Full five-variable model** |
| --- | --- | --- | --- | --- |
| AUC in held-out internal test set | 0.681 | 0.735 | 0.784 | 0.816 |
| 95% CI for AUC | 0.604–0.758 | 0.666–0.804 | 0.716–0.852 | 0.754–0.878 |
| ΔAUC vs. four-variable model | Not applicable | Not applicable | Reference | 0.032 |
| P value vs. four-variable model | Not applicable | Not applicable | Reference | 0.041 |
| Hosmer-Lemeshow P value | 0.192 | 0.266 | 0.438 | 0.510 |
| Calibration-in-the-large | −0.08 | −0.06 | −0.04 | −0.03 |
| Calibration slope | 0.88 | 0.90 | 0.92 | 0.94 |
| Brier score | 0.181 | 0.173 | 0.164 | 0.151 |
| Scaled Brier score | 0.162 | 0.199 | 0.241 | 0.301 |
| Sensitivity | 67.2% | 70.7% | 72.4% | 75.4% |
| Specificity | 64.4% | 69.5% | 73.2% | 75.8% |
| PPV | 48.8% | 54.1% | 58.7% | 62.7% |
| NPV | 76.8% | 80.4% | 83.1% | 85.2% |
| Accuracy | 65.3% | 69.9% | 72.7% | 75.6% |

**Notes:** The four-variable model excluded first-trimester TG and retained pre-gravid BMI, FPG at GDM diagnosis, 1hPG at GDM diagnosis, and first-trimester HDL-C. The full five-variable model additionally included first-trimester TG. All models were fitted in the training set and evaluated in the held-out internal test set. Pairwise AUC comparison between the four-variable model and the full five-variable model was performed using DeLong’s test. TG, triglycerides; BMI, body mass index; FPG, fasting plasma glucose; 1hPG, 1-hour post-load glucose; HDL-C, high-density lipoprotein cholesterol; AUC, area under the receiver operating characteristic curve; PPV, positive predictive value; NPV, negative predictive value.

**Supplementary Table S6.** Sensitivity analysis additionally adjusting for gestational age at outcome lipid measurement

| **Metric** | **Primary model** | **Sensitivity model additionally adjusted for gestational age at lipid measurement** |
| --- | --- | --- |
| AUC in held-out internal test set | 0.816 | 0.814 |
| 95% CI for AUC | 0.754–0.878 | 0.752–0.876 |
| Hosmer-Lemeshow test P value | 0.510 | 0.487 |
| Sensitivity | 75.4% | 74.8% |
| Specificity | 75.8% | 75.2% |
| PPV | 62.7% | 62.1% |
| NPV | 85.2% | 84.9% |
| OR for gestational age at lipid measurement | Not included | 1.06 per week |
| 95% CI for OR | Not applicable | 0.89–1.27 |
| P value for gestational age at lipid measurement | Not applicable | 0.502 |

Notes: The primary model included pre-gravid BMI, FPG at GDM diagnosis, 1hPG at GDM diagnosis, first-trimester TG, and first-trimester HDL-C. The sensitivity model additionally included gestational age at outcome lipid measurement to assess whether residual measurement-time variation within the predefined 32+0 to 36+6-week window influenced model performance. Abbreviations: AUC, area under the receiver operating characteristic curve; CI, confidence interval; OR, odds ratio; PPV, positive predictive value; NPV, negative predictive value; BMI, body mass index; GDM, gestational diabetes mellitus; FPG, fasting plasma glucose; 1hPG, 1-hour post-load glucose; TG, triglycerides; HDL-C, high-density lipoprotein cholesterol.

**Supplementary Table S7.** Sensitivity analyses using alternative triglyceride cut-offs for defining late-pregnancy HTG

| **Metric** | **≥1.7 mmol/L** | **≥2.3 mmol/L primary** | **≥2.5 mmol/L** | **≥2.83 mmol/L** |
| --- | --- | --- | --- | --- |
| HTG incidence | 411/587 (70.0%) | 192/587 (32.7%) | 152/587 (25.9%) | 96/587 (16.4%) |
| AUC in held-out internal test set | 0.781 | 0.816 | 0.807 | 0.789 |
| 95% CI for AUC | 0.712–0.850 | 0.754–0.878 | 0.737–0.877 | 0.704–0.874 |
| Hosmer-Lemeshow test P value | 0.362 | 0.510 | 0.446 | 0.391 |
| Sensitivity | 78.2% | 75.4% | 72.6% | 69.8% |
| Specificity | 68.5% | 75.8% | 77.9% | 80.6% |
| PPV | 83.6% | 62.7% | 53.8% | 40.2% |
| NPV | 62.9% | 85.2% | 88.7% | 91.6% |

Note: All sensitivity analyses were performed within the predefined 32+0 to 36+6-week outcome window. The same five-predictor model structure was used for each outcome definition, and model coefficients were refitted in the training set before evaluation in the held-out internal test set. The ≥2.3 mmol/L threshold was used as the primary outcome definition. Abbreviations: HTG, hypertriglyceridemia; TG, triglycerides; AUC, area under the receiver operating characteristic curve; CI, confidence interval; PPV, positive predictive value; NPV, negative predictive value.

**Supplementary Table S8.** Sensitivity analysis replacing self-reported pre-pregnancy BMI with BMI calculated from first measured antenatal weight

| **Metric** | **Primary model** | **Sensitivity model using first measured antenatal BMI** |
| --- | --- | --- |
| AUC in held-out internal test set | 0.816 | 0.811 |
| 95% CI for AUC | 0.754–0.878 | 0.748–0.874 |
| Calibration-in-the-large | −0.03 | −0.04 |
| Calibration slope | 0.94 | 0.92 |
| Hosmer-Lemeshow P value | 0.510 | 0.462 |
| Brier score | 0.151 | 0.154 |
| Sensitivity | 75.4% | 74.1% |
| Specificity | 75.8% | 75.0% |
| PPV | 62.7% | 61.8% |
| NPV | 85.2% | 84.6% |
| Accuracy | 75.6% | 74.7% |

**Notes:** The sensitivity model replaced pre-gravid BMI with BMI calculated from first measured antenatal weight. The remaining four predictors were unchanged: FPG at GDM diagnosis, 1hPG at GDM diagnosis, first-trimester TG, and first-trimester HDL-C. Both models were fitted in the training set and evaluated in the held-out internal test set.
